# Nocturnal Awakening & Sleep Duration in Veterans with PTSD: An Actigraphic Study

**DOI:** 10.12669/pjms.294.3831

**Published:** 2013

**Authors:** Imran S. Khawaja, Ali M. Hashmi, Joseph Westermeyer, Paul Thuras, Thomas Hurwitz

**Affiliations:** 1 Imran S. Khawaja, MD; 2 Minneapolis VA Medical Center, University of Minnesota, State of Minnesota, USA.; 3 Ali M. Hashmi, MD, Foreign Professor III (HEC), Psychiatry, King Edward Medical University/Mayo Hospital, Lahore.; 4 Joseph Westermeyer, MD, PhD, Paul Thuras, PhD, Minneapolis VA Medical Center, University of Minnesota, State of Minnesota, USA.; 5 Thomas Hurwitz, MD, Minneapolis VA Medical Center, University of Minnesota, State of Minnesota, USA.

**Keywords:** PTSD, Insomnia, Actigraphy, Veteran

## Abstract

***Objective:*** To assess whether awakenings from sleep and sleep duration in Post Traumatic Stress Disorder (PTSD) were related to demography, posttraumatic or depressive symptoms, subjective sleep quality, and daytime sleepiness.

***Methods:*** Sample consisted of 23 veterans with lifetime PTSD and current sleep disturbance not due to apnea or other diagnosable conditions. Data collection included demography, two weeks of actigraphy, Beck Depression Inventory, Posttraumatic Checklist, Clinical Assessment of Posttraumatic Symptoms, Pittsburgh Sleep Quality Index, and Epworth Sleepiness Scale.

***Results: ***The study revealed that awakenings increased with younger age. Variability in awakenings also increased with younger age (p = 0.002). More awakenings were associated with shorter sleep duration.

***Conclusions: ***These paradoxical observations regarding younger age and more awakening may be related to increased sleep symptoms early in the course and then gradual waning of posttraumatic symptoms over time, since awakenings tend to increase with age in normals (rather than decrease, as we observed).

## INTRODUCTION

Sleep disturbances are among the most prevalent symptoms in patients with mental health disorders. Though awakenings and other forms of sleep disturbance are common in Post Traumatic Stress Disorder (PTSD), yet they are not a universal concomitant of PTSD with different investigators having different opinions.^1^^,^^2^ One text on sleep disorders barely mentions PTSD as a cause of sleep disturbance.^3^ Although one study mentions sleep disturbances as a cardinal symptom of PTSD^4^, two studies, one using polysomnography^5^ and the other using actigraphy^6^, failed to demonstrate objective evidence of sleep disturbance in post-trauma survivors. A meta-analysis of 20 studies, comparing polysomnographic sleep data between patients with and without PTSD showed that PTSD patients had increased stage N1, decreased stage N3 and greater stage REM density.^7^ Nonetheless, most combat veterans and rape victims with PTSD whom we studied with a daily life charting instrument reported considerable sleep disturbance.^8^ Other investigators have observed that repetitive awakenings, nightmares, insomnia, and daytime sleepiness pose common problems among those with PTSD.^9^^-^^11^ In a recent study of returning soldiers, insomnia was the most commonly reported symptom and predicted other symptoms of PTSD.^12^ Another study^13^ comprising 191 minority youths who survived Hurricane Katrina, suggested that general sleep disturbance at 24 months predicted PTSD symptom severity at 30 months even after adjusting for PTS symptom severity at 24 months.^13^

On the other hand one theory holds that sleep disturbance in patients with PTSD may primarily be due to co-morbid depression.^14^^,^^15^ Still others opine that the PTSD-insomnia relationship is a complex phenomenon that requires the development of a strong theoretical base before clinical understanding is feasible.^16^

The purpose of this study was to assess whether awakenings and sleep duration bore a relationship to severity of posttraumatic and depressive symptoms in veterans with Posttraumatic Stress Disorder (PTSD) and sleep disturbance. Our earlier actigraphic study demonstrated that veterans with PTSD and sleep disturbance had increased awakenings and shortened sleep duration.^17^ Rationale for the overall study was to expand our understanding of PTSD-related awakenings and short duration of sleep, which cause subjective distress, resist treatment, and can result in alcohol and drug abuse.^18^^-^^20^

We evaluated veterans’ sleep in their normal at-home setting using self-report and actigraphy. This at-home approach removed the reassuring presence of a technician in the adjacent room (a concomitant of most polysomnographic studies) and allowed for a more accurate measure of sleep duration and awakenings throughout a typical two-week period. The actigraph provided data that corresponded to sleep, awakenings, and other indices of sleep.^17^ In order to obtain a sample reflecting common clinical practice, we sampled a mixed group of patients with diverse co-morbid disorders and treatment histories, so as to obtain a sample reflecting common clinical practice. This study tested the following hypotheses:

1. Increased awakenings would be associated with depressive symptoms and posttraumatic symptoms.
2. Sleep duration would be inversely associated with number of awakenings and depressive symptoms, and with posttraumatic symptoms.

## METHODS

***Study Population:*** Study participants consisted of volunteer veterans who were receiving care at the Minneapolis VA Medical Center. In order to be admitted to the study, participants had to have a lifetime diagnosis of Posttraumatic Stress Disorder, a current sleep disturbance and stability of psychiatric disorder. Exclusionary data included obstructive sleep apnea, psychosis, dementia, delirium, and homelessness and current substance use. There were no other restrictions by age, gender, race/ethnicity, medication, or other co morbidity than described above.

Thirty-five study participants were in the original sample. Eight of these 35 study participants met additional exclusion criteria (e.g., active psychosis, homelessness, active substance dependence). One participant did not provide actigraphic data, and so was dropped from the study, leaving 26 study participants. Three of these 26 were dropped due to insufficient baseline and/or termination data for analysis, leaving 23 participants for this final analysis. Study participants received compensation for their time in the study. All the participants were duly informed about the procedure and an informed consent was taken.

***Data Collection:***

Actigraphy: Actigraphs, which detect, measure, and record movement, have shown a high correlation with polysomnographic study of sleep and wakefulness.^21   ^ Correlations between actigraphy and polysomnography for total sleep time have been high, in the range of 0.97.^22^ Correlations for sleep onset latency and awakenings have been less consistent, with a tendency for actigraphy to under-record “quiet awakenings” detected with polysomnography.^22^,^23^

Patients wore the actigraphs on the non-dominant wrist 24 hours per day, except for period when in water (e.g., showering, bathing). The actigraphs used were octagonal Basic, Ultra, and Advanced models manufactured by Ambulatory Monitoring, Inc (731 Saw Mill River Road Ardsley, NY 10502-0609) each providing the same measures. The devices were worn throughout the study period, providing seven-to-fourteen 24-hour cycles per participant; mean cycles per participant was 11.5 nights. Data from the actigraphs were down-loaded for analysis using Action W software from Ambulatory Monitoring, Inc. Actigraphy-based data used for this analysis included mean sleep time per participant (with its standard deviation), and mean number of awakenings per night (with its standard deviation).

Study participants were asked to wear the actigraph for fourteen nights. Seven study participants complied fully with instructions, providing 14 nights/days of actigraphic data. The remaining 14 study participants missed some days of data collection, often due to their forgetting to put the actigraph back on after bathing.

***Pittsburgh Sleep Quality and Epworth Sleepiness Scale:*** The Pittsburgh Sleep Quality Index (PSQI) is a nineteen item self-rated assessment questionnaire which appraises sleep quality and sleep disturbances.^24^ The Epworth Sleepiness Scale (ESS) is a measure used to determine daytime sleepiness through self-rating on eight items^25^; participants rate how likely they would be to fall asleep in eight different real-life circumstances. These instruments were included to compare and contrast subjective sleep reports with the objective actigraphy data.

***Beck Depression Inventory (BDI), Posttraumatic Stress Disorder Checklist (PCL), and Clinician Administered PTSD Scale (CAPS):*** The Beck Depression Inventory (BDI) is a twenty-one item multiple choice self-reported questionnaire that assesses current depressive symptoms.^26^ The Posttraumatic Stress Disorder Checklist (PCL) is a twenty-one item self-reported questionnaire that assesses current posttraumatic symptoms.^27^ The BDI and PCL were administered before and after the period of actigraphic data collection. The ratings used for this study consisted of an average of the baseline and terminal ratings for both the BDI and PCL.

The Clinician Administered PTSD Scale (CAPS) is a structured interview-based measure comprised of 30-items that correspond to the DSM-IV criteria for PTSD.^28^ The CAPS can be used to make a current (past month) or lifetime diagnosis of PTSD. Participants were administered the CAPS at baseline to assist in determining qualification into the study. The CAPS was administered at the termination of the study. To qualify for the study, participants had to have a lifetime diagnosis of PTSD.

***Data Analysis: ***The statistical analysis was done using SPSS 15.0. For categorical measures-versus-actigraphy (e.g., gender), we employed the t test, two tailed. Skewness was 1 or less for all of these continuous data. With continuous variables (e.g., age), the Pearson correlation coefficient was used. Level of significance was set at .05. A regression analysis was also undertaken as described below.

## RESULTS

***Awakening: ***Mean actigraphic awakenings per veteran ranged from 4.1 to 18.4 awakenings per night, with a group mean of 9.3 awakenings and a standard deviation of 3.8. Median number of awakenings was 9.4 per night. Distribution of awakenings was as follows:

- 4.0 to 5.9 awakenings: 6 participants;
- 6.0 to 7.9 awakenings: 2 participants;
- 8.0 to 9.9 awakenings: 6 participants
- 10.0 to 11.9 awakenings: 5 participants;
- 12.0 to 13.9 awakenings: 2 participants;
- 14 awakenings or more: 2 participants.

As with sleep duration, number of awakenings also varied over time within the same veteran as well as across veterans. The person with the most consistent awakenings had a standard deviation of 2.7, and the person with the greatest variability in awakenings had a standard deviation of 11.1. Greater variability in awakenings was associated with a higher mean number of awakenings (r = + .56, p = .006) and a lower mean duration of sleep (r = - .53, p = .009).

***Sleep Duration:*** Mean actigraphic sleep duration per veteran ranged from 75.0 minutes to 520.6 minutes (1.3 to 8.7 hours), with a group mean of 352.7 minutes (5.9 hours) and a standard deviation of 119.9 minutes (2 hours). Measures ran for 24 hours, noon-to-noon. Distribution of study participants by mean sleep duration per 24-hours ranged widely, as follows:

1.0-1.9 hours: 1 participant;

2.0-2.9 hours: 1 participant;

3.0 to 3.9 hours: 3 participants;

4.0 to 4.9 hours: 2 participants;

5.0 to 5.9 hours: 4 participants;

6.0 to 6.9 hours: 5 participants;

7.0 to 7.9 hours: 3 participants;

8.0 to 8.9 hours: 4 participants.

Those who were sleeping in the range of 7.0 to 8.9 hours complained of awakening at night, not awakening refreshed from sleep in the morning, daytime sleepiness, and/or nightmares. The most consistent sleeper showed a standard deviation for sleep duration of only 19.1 minutes, while the sleeper with the most variability showed a standard deviation of 234.1 minutes. For the entire group, sleep variability for each individual was approximately 2 hours. Sleep duration and number of awakenings were not correlated with each other (r = - .33, p = .13).

***Demography: ***The mean age of the participating population was 52.8 ± 10.3 with the range of 31 to 86 years. As shown in Table-I, mean duration of sleep per night did not differ by gender, marital status, education employment, or residence. The mean sleep duration was 50 to 60 minutes longer for women, for people who were married, for those not employed, and for participants living with family.

Younger age was associated with more awakenings (p = .02); see Table-II. Education, marital status, employment, and residence were not associated with awakening. Since more awakenings were associated with greater variability (i.e.; larger standard deviation) in awakenings, we also compared age and variability in awakenings. Greater variability in awakening was also associated with younger age (r = - .61, p = .002).

As shown in Table-II, younger age showed a borderline correlation with shorter duration of sleep (p = .07).

***Self-Rated Sleep Quality and Excessive daytime sleepiness: ***Number of awakenings was not correlated with either the Pittsburgh Sleep Quality Index or the Epworth Sleepiness Scale. Longer duration of sleep on actigraphy showed a trend for a correlation with higher scores on the Pittsburgh Sleep Quality (p = .08) but not on the Epworth Sleepiness Scale. (Table-II).

***Symptom Scales: ***Number of awakenings was not associated with the Beck Depression Inventory, the Posttraumatic Checklist, or the Clinician Administered PTSD Scale. Duration of sleep was also not associated with the Beck Depression Inventory, the Posttraumatic Checklist, or the Clinician Administered PTSD Scale.(Table-II).

***Regression Analysis: ***In order to assess the combined effect of age and mean number of awakenings on variability of awakenings, we conducted a linear regression analysis; see Table-III. Age was inversely related to awakenings (p = .03). Number of awakenings was not independently related to variability in awakenings.

**Table-I:** Actigraphic findings versus demographic characteristics

*Characteristic (n = 23)*	*Mean*	*Stan. Dev.*	*Statistics*
*Mean nightly awakenings per person*			
Gender male (n = 20) female (n = 3)	9.8 awakens 5.6 awakens	3.7 2.2	t = 1.93 df = 21 p = .07
Marital status, current married (n = 11) other (n = 12)	10.3 awakens 8.3 awakens	4.5 2.8	t = 1.26 df = 21 p = .22
Employment, current employed (n = 7) other (n = 16)	10.2 awakens 8.9 awakens	4.1 3.6	t = 0.78 df = 21 p = .44
Residence, current family (n = 13) other (n = 10)	10.2 awakens 8.1 awakens	4.2 2.9	t = 1.39 df = 21 p = .16
*Mean nightly sleep per person in minutes*			
Gender male (n = 20) female (n = 3)	345.3 min. 402.4 min.	124.4 83.2	t = 0.76 df = 21 p = .45
Marital status, current married (n = 11) other (n = 12)	380.0 min. 327.7 min.	115.4 123.3	t = 1.05 df =21 p = .31
Employment, current employed (n = 7) other (n = 16)	310.9 min. 371.0 min.	133.4 113.1	t = 1.11 df = 21 p = .28
Residence, current family (n = 13) other (n = 10)	375.6 min. 323.0 min.	121.8 116.7	t = 1.04 df = 21 p = .31

**Table-II:** Correlations of actigraphic findings with demography, self-rated sleep scales, and self-rated symptom scales

*Characteristic*	*Actigraphic Findings*	
	*Mean Awakenings *	*Mean Sleep Duration*
Demography (n = 23)		
Age	r = - .48, p = .02	r = + .39, p = .07
Education (years)	r = + .25, p = .26	r = - .01, p = .97
Self-Rated Sleep Scales (n = 22)		
Pittsburg Sleep Quality	r = - .23, p = .30	r = +.37, p = .08
Epworth	r = - .32, p = .14	r = + .13, p = .55
Self-Rated Symptoms Scales (n = 23)		
Beck Depression Inventory	r = - .003, p = .99	r = + .06, p = .77
Posttraumatic Checklist, total score	r = ,- .13 p = .56	r = + .06, p = .79
Psychiatrist-Rated Clinical Assessment for Posttraumatic Symptoms (n = 20)		
CAPS score, current	r = - .33, p = .16	r = + .01 p = .98

**Table-III:** Linear Regression Analysis: Predicting the variability in number of awakenings per night

Variables	B	S.E.	Beta	t	Signif.	95% CI (B)
Constant	9.52	2.97		3.21	.004	.3.33-15.72
Age	-.10	.04	-.45	2.40	.03	-.19 to -.01
Awakenings, mean	.20	.11	.34	1.83	.08	-.03 to -.43

## DISCUSSION

***Objective Findings of Sleep Disturbance: ***Actigraphy revealed a high rate of awakenings in this group, with no one experiencing less than four awakenings per night on average. Twenty out of 23 study participants (or 93%) had more than 5 awakenings per night on average. As reported in our earlier study^17^, participants reported only a fraction of the awakenings seen on actigraphy, indicating that they were experiencing more sleep disturbance than they reported. Actigraphy also revealed that a high percentage of study participants showed objective evidence of reduced sleep duration. Nineteen out of 23 participants (or 83%) slept for less than eight hours per night during the observation period. About half of them (11 out of 23) slept less than six hours per night on average, and about one-third (7 out of 23) slept less than five hours per night on average.

Variability in number of awakenings and variability in sleep duration were notable findings. Only a few people showed consistent awakenings and sleep duration. Wide fluctuations from one night to the next were the norm. This considerable variability itself poses a major problem for these patients, who have difficulty reliably predicting how disrupted their sleep is apt to be on any one night. The variability also comprises a major challenge for clinicians, whose ministrations may be difficult to assess in light of such wide variations even over a brief two-week period.

Unlike other studies that have included a diversity of participants with PTSD^5^^,^^6^^,^^16^^,^^29^^,^^30^, our study selected for people with sleep disturbances and a stable course of PTSD. Thus, the sample method as well as the means of data collection (i.e., at-home actigraphy) favored the detection of objective sleep findings, as compared to other sample approaches (e.g., all people with PTSD regardless of sleep complaints) and data collection methods (e.g.; polysomnography in an institutional setting).

***Association of Sleep Findings with Demographic and Clinical Findings: ***The only findings to reach significance were (1) younger age and more awakenings on univariate comparison (p = .02), (2) younger age and greater variability in awakenings on regression analysis (p = .03), and (3) shorter duration of sleep and greater variability in awakenings on univariate comparison (p = .009). 

One explanation for the inverse relation of age and more sleep disruption could be greater temporal proximity of younger veterans to their criterion A traumatic experiences. Other investigators have observed a gradual diminution of symptoms during the course of PTSD in many, but not all cases.^31^^-^^34^ Thus, the improved sleep with age may be due to gradual clinical improvement over time.

***Relevance to Pakistan: ***The Pakistan Army has been in a deadly ‘War on Terror’ since about 2004. According to some estimates, the Army has lost over three thousand troops, equivalent to two full brigades in this conflict.^35^ This count does not include soldiers who have suffered significant injuries or other incidents.^36^^,^^37^ which would predispose them to PTSD and its attendant complications. In spite of this, there are no published studies on the incidence, prevalence or risk factors of PTSD in Pakistani soldiers. This study underlines the urgent need for such studies to be conducted. 

***Limitations of the Study: ***A small sample size could have increased the chance of a false negative finding. Since only three women were included in the sample, true gender differences may have been overlooked. The heterogeneity of the sample in terms of medication and co morbidity may have undermined trends that could be recognized in a more homogeneous group. Comparison with one or more control groups (e.g., veterans with PTSD but not sleep disturbance, veterans with comparable sleep disturbance but not PTSD) may have pointed up the special characteristics of this group. For exclusion criteria of OSA, clinical history was used instead of a formal PSG. 

## Author’s Contributions:

Imran S. Khawaja was involved in conception and design of the study, data analysis and interpretation, manuscript writing, and final approval. Ali Madeeh Hashmi contributed to study conception and design, data acquisition, analysis and interpretation, and drafting the article. Joseph Westermeyer was involved in data acquisition, statistical analysis and interpretation, along with drafting and editing the manuscript. Paul Thuras helped with data interpretation, manuscript writing and final revision. Thomas Hurwitz contributed to study design, data collection, entry and statistical analysis.
